# Detection of Early Glaucomatous Damage in Pseudo Exfoliation Syndrome by Assessment of Retinal Nerve Fiber Layer Thickness

**DOI:** 10.4103/0974-9233.56228

**Published:** 2009

**Authors:** Maha M. Mohamed

**Affiliations:** Department of Ophthalmology, Faculty of Medicine, kasr el Aini Hospital, Cairo University

**Keywords:** Glaucoma, Optical Coherence Tomography, Pseudo Exfoliation Syndrome, Retinal Nerve Fiber Layer Thickness

## Abstract

**Purpose:**

To detect early glaucomatous changes in pseudo exfoliative patients with normal intraocular pressure (IOP), visual field and optic nerve head appearance; by measuring retinal nerve fiber layer (RNFL) thickness using optical coherence tomography (OCT).

**Design:**

A prospective observational case-control study.

**Participants:**

Twenty non-glaucomatous (normal IOP, fundus and visual field) pseudo exfoliative patients and 20 age matched healthy control subjects.

**Materials and Methods:**

The RNFL thickness (global and four quadrants) was assessed using combined imaging system OTI (OCT/SLO) and compared with age matched normal control subjects.

**Results:**

The RNFL in patients with pseudo exfoliation syndrome (PXS) was significantly thinner in all quadrants except the nasal quadrant compared to the control group (p less than 0.05).

**Conclusion:**

Measurement of RNFL thickness by OCT is useful in detecting early RNFL damage which in turn provides clinically relevant information in detecting early glaucomatous changes in pseudo exfoliative patients.

## INTRODUCTION

Pseudo exfoliation syndrome (PXS) is an age-related, generalized disorder of the extracellular matrix characterized by production and progressive accumulation of an abnormal extracellular pseudo exfoliative material in many intra- and extraocular tissues. The presence of this material causes important changes to the cornea, angle, lens and zonules.^1^

Active involvement of the trabecular meshwork in this characteristic matrix process may lead to secondary open angle glaucoma (pseudo exfoliative glaucoma (PXG)) in 40-60% of the patients.^2^

PXG represents a relatively severe and progressive type of glaucoma, with a generally poor prognosis due to high intraocular pressure levels, great pressure differences between the two eyes and fluctuations in the diurnal pressure curve.^3^

The primary cause of chronic pressure elevation appears to be local production of pseudo exfoliation (PEX) material by trabecular meshwork and Schlemm's canal cells with subsequent degenerative changes of Schlemm's canal and juxta canalicular tissues. Additional pathogenetic factors contributing to pressure increase include pronounced melanin dispersion, increased protein concentrations of the aqueous humor, vascular factors, and connective tissue alterations of the lamina cribrosa.^2^

As RNFL damage may present before any detectable visual field loss, IOP elevation and optic nerve head defects as an early clinical sign of open-angle glaucoma; early glaucomatous changes in PXS, despite normal IOP, optic nerve appearance and visual field; can be detected by measuring RNFL thickness using OCT.^4^

### Aim

To investigate the possibility of detection of early glaucomatous damage in PXS with normal IOP, visual field and optic nerve head appearance by assessing the RNFL thickness using OCT.

## MATERIALS AND METHODS

A prospective observational case control study was carried out on 40 eyes; 20 non-glaucomatous (normal IOP, fundus and visual field) eyes with PXS (group A) and 20 age matched normal control subjects (group B). The study was conducted from February to December 2008 in the Ophthalmology Department of Kasr El Einy Hospital.

### Inclusion criteria

Pseudo exfoliative patients (clinically diagnosed) and normal age matched control subjects with:

- Normal optic nerve head appearance (with C/D ratio less than or equal to 0.3 and asymmetry less than or equal to 0.2 between fellow eyes) and normal retina.
- Normal intraocular pressure (IOP less than or equal to 21 mmHg with normal diurnal variation and difference less than or equal to three mmHg between fellow eyes).
- Normal visual field test. It is considered abnormal if any two of the following three criteria were met on at least two consecutive visual field tests:

- Glaucoma hemi-field test outside normal limits,
- A cluster of three or more non-edge points in a location typical for glaucoma all of which are depressed on the pattern deviation plot at a *p* less than five per cent level and one of which is depressed at a *p* less than one per cent level, three corrected pattern standard deviation *p* less than five per cent.
- Clear media with no history of any systemic or ocular disease, surgery and trauma.

BCVA for all patients is of least 0.9 with refractive error between plus three and minus three dpt sphere and not exceeding two dpt cylinder.

### Exclusion criteria

1. Glaucomatous patients or family history of glaucoma.
2. Media opacity interfering with visualization and OCT images capturing such as corneal opacity or dense cataract.
3. History of any ocular diseases.
4. History of any systemic diseases such as diabetes mellitus and hypertension.
5. History of previous intraocular surgery.
6. Previous ocular trauma.
7. Retinal pathology.

After informed consent was signed, all participants underwent the following examinations: Visual acuity (BCVA), slit lamp biomicroscopy (to detect signs of PXS such as pseudo exfoliative material at the pupillary border, anterior lens capsule or angle, sphincteric atrophy and lens sublaxation), Goldmann applanation tonometry, gonioscopy, ophthalmoscopy, automated refraction and Humphrey (central 24-2 threshold) visual field examination.

The RNFL thickness was assessed in all patients in the Ophthalmic Diagnostic and Laser Unit (DLU) using combined imaging system; OTI Spectral OCT/SLO (Ophthalmic Technologies Inc. (OTI), Toronto, Ontario, Canada). A circular OCT Scan with a diameter of 3.4mm was placed around the optic nerve while the location was observed on the confocal SLO image (topographic map of the optic nerve area using 128 longitudinal scans over a five mm area) to ensure proper positioning in relation to the optic nerve. The RNFL thickness map was displayed along with its ratio to normative RNFL thickness.

The global and four-quadrant average RNFL thickness data (temporal, superior, nasal and inferior) was collected and compared in both groups. The results were reported as mean values plus/minus SD. Data was statistically analyzed. An unpaired *t* -test was used to calculate the *p* value between the study and the control group. Values of *p* less than 0.05 were considered statistically significant.

### RESULTS

Forty eyes of 40 patients were included in the study; 20 eyes with non-glaucomatous PXS (group A) and 20 age- matched free control subjects (group B).

Patient age ranged from 45-65 years in both groups with a mean of 56.2 plus/minus 5.4 SD in group A and 54.6 plus/minus 5.7 SD in group B. There were 12 (60%) males and eight (40%) females in group A and nine (45%) males and 11 (55%) females in group B. The difference between both groups was statistically insignificant.

In group A the mean IOP was (15.1 plus/minus 1.7 SD) and the visual field mean deviation was (0.92 plus/minus 0.86 SD), while in group B the mean IOP was (14.2 plus/minus 1.07 SD) and the visual field mean deviation was (0.89 plus/minus 0.9 SD). The difference between both groups was statistically insignificant (*p* greater than 0.05). This suggests the relationship between IOP and RNFL damage in PXS in this cohort and within the parameters of the study design is not clear and a larger study is needed to further investigate this unique observation. The demographic and inclusion data of patients is summarized in [Table 1].

**Table 1:** Patients' demographic and inclusion data

	PXS group (A)	Control group (B)
No. of patients	20	20
Age (Mean ± SD)	56.2 ± 5.4	54.6 ± 5.7
Sex(%)	(60) ♂	(45) ♂
	(40) ♀	(55) ♀
IOP (Mean ± SD)	15.1 ± 1.7	14.2 ± 1.07
Mean deviation (VF testing)	0.92 ± 0.86	0.89 ± 0.9

In each eye, average RNFL thickness measurements were obtained in temporal, superior, nasal, and inferior quadrants. A single index of average RNFL thickness throughout 360° also was obtained. The RNFL thickness parameters (μm)(mean and SD) measured during the study in both groups are presented in [Table 2]

**Table 2:** RNFL thickness parameters (mm) (mean and SD) global and for each quadrant in (group A and B)

RNFL Quadrants	PXS group (A) (Mean ± SD)	Control group (B) (Mean ± SD)	*P* value
Temporal	60.65 ± 7.85	72.45 ± 8.15	<0.05
Superior	112.9 ± 14.05	130.1 ± 9.33	<0.05
Nasal	67.35 ± 11.1	71.7 ± 8.16	>0.1
Inferior	105.2 ± 11.63	122.6 ± 11.81	<0.05
Global average	86.52 ± 19.7	99.21 ± 20.21	>0.05

**Figure 1:**
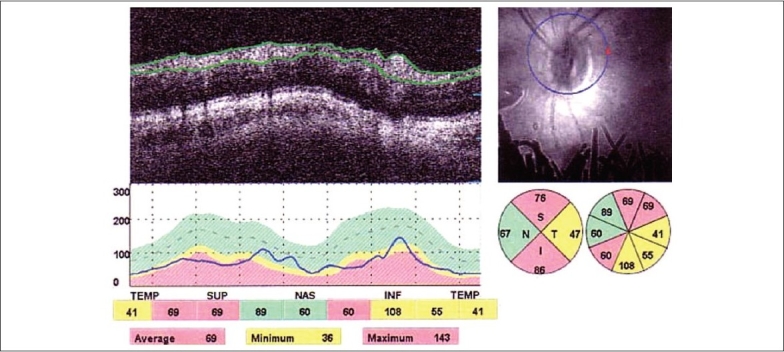
OCT scans around optic nerve head with analysis of the RNFL thickness in a pseudo exfoliative eye showing diminished thickness in all quadrants except nasal quadrant

In (group A), the values of RNFL thickness measurement in all quadrants were low compared to (group B). The average overall RNFL thickness was (86.52 plus/minus 9.7) in (group A) and (99.21 plus/minus 20.21) in (group B) with no quiet statistically significant difference between both groups (*P* greater than 0.05) [Figure 1]

**Figure 2:**
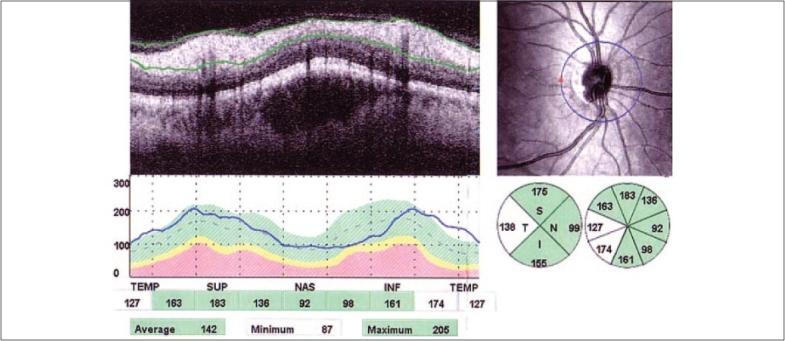
OCT scans around optic nerve head in a normal eye with double peak curve of RNFL thickness

In (group B); normal eyes revealed a characteristic double-hump pattern (double peak curve) with RNFL thickness peaks in the superior and inferior quadrants (thickest) and troughs in the temporal and nasal (thinnest) [Figure 2]. The distribution of the RNFL measured by OCT is analogous to the normal histological distribution.

**Figure 3:**
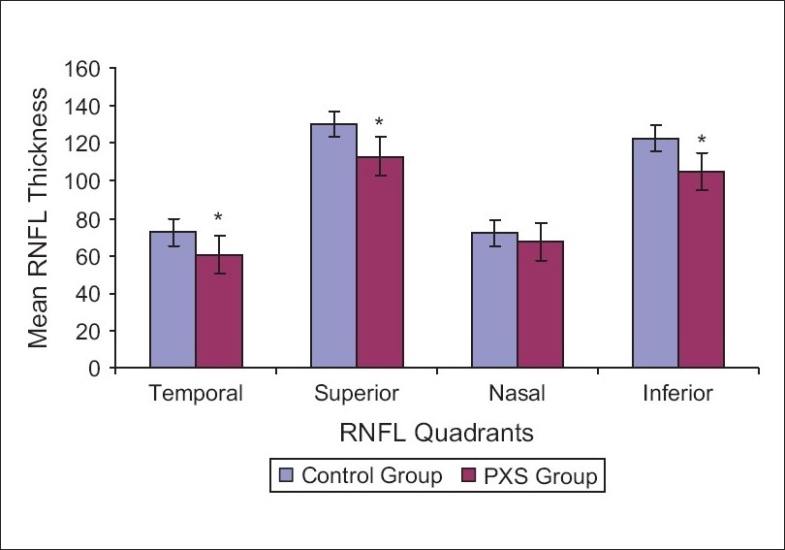
Comparison between RNFL thickness in all quadrants in both groups

As regards quadratic RNFL thickness measurement in both groups; eyes with PXS showed significantly thinner RNFL compared to normal control eyes in all quadrants (*p* less than 0.05) except the nasal quadrant (*p* greater than 0.1) [Figure 3].

### DISCUSSION

Secondary chronic open-angle glaucoma associated with PXS accounts for approximately 25% of all glaucoma and represents the most common identifiable cause of glaucoma overall.^3^

The exact mechanism of progression of PXS to PXG remains unknown. The higher IOP in PXS, vascular change with alteration of ocular blood flow or the pseudo exfoliation material itself may be the main factors in causing RNFL damage.^5^

The RNFL defect, which may be presented before any detectable IOP elevation or optic nerve head and visual field damage, is a main sign of early glaucomatous damage. This suggests that visual field testing; fundus image and IOP may not be sensitive to detect this early damage.^4^

The high diagnostic accuracy of the Spectral OCT/SLO allows for rapid, reproducible OCT scanning of the RNFL thickness and monitors changes in thickness for the detection of early glaucoma.^6^

The aim of this study is to detect early RNFL damage in pseudo exfoliative patients without glaucoma by measuring RNFL thickness using spectral OCT/SLO and comparing the results with age matched healthy control subjects.

This study demonstrated the mean overall RNFL thickness in both groups which was lower in group A than group B but with statistical insignificance difference between them.

The RNFL thickness in normal subjects (group B); was normally thicker in the superior and inferior, thinner in the temporal and lowest in the nasal quadrants; however in group A; there were significant differences in RNFL thickness among the four quadrants except for the nasal quadrant compared to the control group. This could be explained by the fact that higher axonal density and higher proportion of large fibers occupies the supero and inferotemporal portions of the optic nerve head compared to the nasal portion. These fibers are in addition; most susceptible to early glaucomatous damage.

Yüksel *et al*. assessed the RNFL thickness in patients with unilateral PXS without glaucoma and their normal fellow eyes using Stratus OCT- 3. The RNFL in patients with PXS were significantly thinner than controls in all quadrants except the nasal quadrant with regard to quadratic and segmental analysis (*p* less than 0.05). This RNFL loss was apparent at 7, 10 and 11 o'clock of the PXS eyes with regard to clock hour position(*p* less than 0.05). In the fellow eyes, no significant difference in RNFL measurement was found except the temporal quadrant when compared with the controls.^5^

In the early stage of PXG; RNFL defect usually progresses to affect mainly local areas in the superior and/or inferior pole. However, with more progression of disease it becomes more extensive and shows diffuse and combination RNFL defects.

Liu *et al.* investigated image characteristics and thickness of RNFL in 83 normal eyes and 83 patients with primary open angle glaucoma with different stages using OCT. They documented a significant difference in RNFL thickness among the four quadrants and the mean overall RNFL thickness except for the nasal quadrant in the early POAG. It was seen that more severe the glaucoma, thinner the RNFL thickness.^4^

Asaoka *et al*. reported also a significant decrease in RNFL thickness measurement in the superior (*p* less than 0.05), superotemporal and inferotemporal sectors (*p* less than 0.01); in early glaucomatous eyes with normal perimetric visual fields and SLO compared to the age matched control subjects.^7^

Sihota *et al.* evaluated the role of (OCT) in detecting differences in peripapillary RNFL thickness among normal and glaucomatous eyes and also among different severities of glaucoma. The average RNFL in control subjects, early glaucoma, moderate glaucoma, severe glaucoma, and blind glaucoma were 102.30 plus/minus 10.34, 77.68 plus/minus 15.7, 66.07 plus/minus 15.5, 53.65 plus/minus 14.2, and 44.93 plus/minus 4.95 micron, respectively. With a significant difference in all RNFL thickness parameters between normal and all glaucoma subgroups (*p* less than 0.001).^8^

### CONCLUSION

PXS without glaucoma may be associated with a thinner RNFL compared with those of age-matched normal subjects. This provides evidence that structural damage preceeds functional loss and that early RNFL thickness assessment using OCT is important in the early diagnosis, intervention and follow-up of pseudo exfoliation glaucoma.
